# Research on the Mechanism of Growth of *Codonopsis pilosula* (Franch.) Nannf. Root Responding to Phenolic Stress Induced by Benzoic Acid

**DOI:** 10.3390/ijms252011007

**Published:** 2024-10-13

**Authors:** Yantong Ma, Lei Ma, Ling Xu, Ruonan Wei, Guiping Chen, Junhong Dang, Zhen Chen, Shaoying Ma, Sheng Li

**Affiliations:** 1 College of Life Sciences and Technology, Gansu Agricultural University, Lanzhou 730070, China; mqdxtl@126.com (Y.M.); xul@st.gsau.edu.cn (L.X.); weirn@st.gsau.edu.cn (R.W.); djh18394144667@163.com (J.D.); 18139956232@163.com (Z.C.); 2 College of Horticulture, Gansu Agricultural University, Lanzhou 730070, China; malei@st.gsau.edu.cn; 3 College of Agronomy, Gansu Agricultural University, Lanzhou 730070, China; chengp@gsau.edu.cn; 4 Laboratory and Practice Base Management Center, Gansu Agricultural University, Lanzhou 730070, China; 5 State Key Laboratory of Aridland Crop Science, Gansu Agricultural University, Lanzhou 730070, China

**Keywords:** *C. pilosula*, benzoic acid, lobetyolin, transcriptomics, soil gas chromatography-mass spectrometry (GC-MS), chemosensory autotoxicity

## Abstract

Soil autotoxic chemosensory substances have emerged as the predominant environmental factors constraining the growth, quality, and yield of *Codonopsis pilosula* in recent years. Among a vast array of chemosensory substances, benzoic acid constitutes the principal chemosensory substance in the successive cultivation of *C. pilosula*. However, the exploration regarding the stress exerted by benzoic acid on the growth and development of *C. pilosula* remains indistinct, and there is a scarcity of research on the mechanism of lobetyolin synthesis in *C. pilosula*. In the current research, it was discovered that exposure to benzoic acid at a concentration of 200 mmol/L conspicuously attenuated the plant height, root length, total length, fresh weight, root weight, root thickness, chlorophyll content, electrolyte osmolality, leaf intercellular CO_2_ concentration (C_i_), net photosynthesis rate (P_n_), transpiration rate (T_r_), and leaf stomatal conductance (G_s_) of *C. pilosula*. Benzoic acid (200 mmol/L) significantly enhanced the activity of root enzymes, including superoxide dismutase (SOD), malondialdehyde (MDA), and peroxidase (POD), as well as the accumulation of polysaccharides and lobetyolins (polyacetylene glycosides) in the roots of *C. pilosula*. In this study, 58,563 genes were assembled, and 7946 differentially expressed genes were discovered, including 4068 upregulated genes and 3878 downregulated genes. The outcomes of the histological examination demonstrated that benzoic acid stress augmented the upregulation of genes encoding key enzymes implicated in the citric acid cycle, fatty acid metabolism, as well as starch and sucrose metabolic pathways. The results of this investigation indicated that a moderate amount of benzoic acid could enhance the content of lobetyolin in *C. pilosula* and upregulate the expression of key coding genes within the signaling cascade to improve the resilience of *C. pilosula* lobetyolin against benzoic acid stress; this furnished a novel perspective for the study of *C. pilosula* lobetyolin as a potential substance for alleviating benzoic acid-induced stress.

## 1. Introduction

*Codonopsis pilosula* [(Franch.) Nannf.], also known as *Chuan Codonopsis* [*Codonopsis pilosula* Oliv.] and *Codonopsis pilosula* [Nannf var. modesta (Nannf.) L. T. Shen] [1], demonstrates a plethora of pharmacological actions encompassing modulation of blood sugar levels, facilitation of hematopoietic functionality, antihypertensive attributes, anti-hypoxic capabilities, fatigue resistance, immune potentiation, retardation of aging, regulation of gastric motility, and ulcer prophylaxis [2]. The cultivation domain for *C. pilosula* in China encompasses 267 counties across 22 provinces; prominently produced in Lintao County within Dingxi City in Gansu Province is the Gansu white-striped variety, which manifests substantial economic benefits [3].

However, the restricted expanse of arable land in conjunction with enhanced economic returns has exacerbated the preponderance of continuous cropping impediments in *C. pilosula*. Amongst these impediments, chemosensory and autotoxic effects exert a substantial influence on the growth, yield, and quality indices of *C. pilosula*. The causative factors for crop failure encompass alterations in the physicochemical properties of the soil, shifts in soil microbial diversity, and chemosensory autotoxicity. Chemosensory autotoxicity is a ubiquitous phenomenon in nature, and constitutes a chemical adaptive mechanism employed by plants to contend with environmental stressors [4]. Despite the considerable medicinal worth of *C. pilosula*, the practice of continuous cultivation gives rise to a considerable accumulation of phenolic acids (e.g., parahydroxybenzoic acid (pHBA), ferulic acid, benzoic acid, cinnamic acid, and vanillic acid) within the soil milieu; this accumulation adversely affects both the growth and productivity of *C. pilosula*. As a consequence, this issue has attracted considerable attention from both researchers and agricultural practitioners [5].

The effect of benzoic acid on the antioxidant enzyme system in plants is significant. In tomato seedlings, the application of benzoic acid and cinnamic acid disrupts the original balance of protective enzymes in the root system, resulting in membrane lipid peroxidation [6]. Benzoic acid treatment significantly reduces SOD activity in faba bean roots and leaves while increasing MDA content [7]. The accumulation of ROS can trigger oxidative stress, disrupting cell homeostasis, inhibiting cellular processes, and even leading to DNA damage and protein oxidation. To cope with oxidative damage, plant cells activate a ROS scavenging system consisting of various antioxidant enzymes such as superoxide dismutase (SOD), POD, MDA, APX, as well as non-enzymatic antioxidants such as glutathione and ascorbic acid. Benzoic acid can significantly inhibit root growth and reduce physiological activity in faba bean seedlings [8,9], *C. pilosula* seedlings [10], tobacco [11], peanuts [12], and Chinese fir trees [13]. Stress also leads to an increase in lignin content and suberin deposition in faba bean roots, exacerbating destruction of their cell defense systems and tissue structure [14,15,16]. Benzoic acid stress has chemosensory effects on membrane lipid peroxidation regulation, osmotic adjustment, and root exudates from Chinese fir trees; low concentrations have inhibitory effects, while high concentrations promote them. These changes may reflect how plants adjust their physiological metabolism pathways to cope with environmental pressures when faced with benzoic acid stress [17]. The above indicates that there is currently a lack of systematic research on the physiological metabolic impact that benzoic acid stress has on *C. pilosula* growth physiology or photosynthesis.

Lobetyolin, a prominent bioactive constituent found in *C. pilosula*, exhibits antioxidant, anti-inflammatory, and immunopotentiating effects. It plays a crucial role as a pharmacological component of this plant [18]. However, limited information is available regarding the biosynthesis of lobetyolin from *C. pilosula*. Previous literature reviews have indicated that lobetyol acts as a precursor for the synthesis of lobetyolin due to its conjugated triple bond, which makes it susceptible to reactions such as addition and oxidation [19]. Notably, members of the Asteraceae, Umbelliferae, Pentacarpaceae, and Solanaceae families are known to synthesize polyalkynyl fatty acid-related secondary metabolites using crepenynic acid (9(Z)-octadecen-12-ynoic acid) as a precursor fatty acid [20]. In daisy Crepis alpina 7, different isoforms of microsomal acyl-lipid-linked fatty acid desaturase introduce an alkyne bond at the Δ12 position of linoleic acid, leading to crepenynic acid production [21]. β-D-glucopyranosyl phenolic glycosides exhibit biological activities such as antiviral and antitumoral properties [22]. β-D-glucopyranosyl phenolic glycosides represent partially substituted derivatives of phenolic compounds that are linked to pyranose derivatives via glycosidic bonds [23]. The predominant source of β-D-glucopyranosyl phenolic glycosides is UDP-glucose, which can be obtained through multiple biosynthetic routes, including direct conversion from UDP-galactose or UDP-xylose [23]. Through literature review, it is known that the glycosides synthesized by *C. pilosula* lobetyolin are derived from β-D-glucose originating from glycolysis/glycolytic metabolism [24]. A dearth of systematic and comprehensive studies and reports persists regarding the synthesis of lobetyol in *C. pilosula*. The molecular regulatory mechanism accountable for the accumulation of lobetyolin in benzoic acid-stressed *C. pilosula* remains elusive. The influence of lobetyol on *C. pilosula* subjected to abiotic stresses has been sparingly reported. In this research, we explored the physiological, biochemical, and photosynthetic responses of *C. pilosula* under benzoic acid stress and analyzed the root transcriptome of *C. pilosula* to illuminate the synthesis mechanism of lobetyolin in *C. pilosula* and the potential mechanism by which lobetyolin counters benzoic acid stress.

## 2. Results

### 2.1. GC-MS Analyses of Rhizosphere Soil Extracts of C. pilosula

The rhizosphere soil extracts of *C. pilosula*, obtained through ethyl acetate extraction after one to four years of continuous cultivation, were subjected to gas chromatography-mass spectrometry (GC-MS) analysis for compositional characterization. The results are presented in Table 1. A total of 13 compounds were identified in the rhizosphere soil extracts during the cultivation periods, including 2 siloxanes, 3 amines, 1 carbamate, 1 aliphatic diol, 4 types of acetyl compounds, 1 fatty acid, 2 thiols, 1 carboxylic acid, 1 chloropropanol compound, and phenolic acids. Benzoic acid was identified as a chemosynthetic substance and suggested for addition to potted plants based on relevant literature review.

### 2.2. Phenotypic Responses of C. pilosula to Different Concentrations of Benzoic Acid

**Figure 1:**
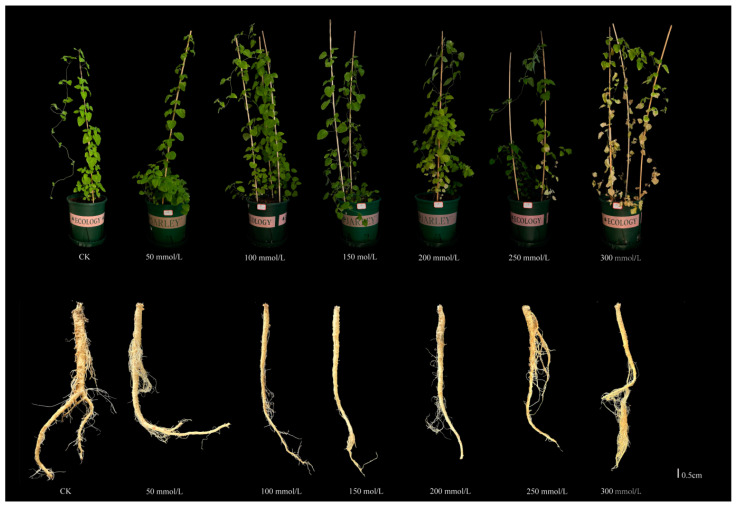
Phenotypic manifestations of *C. pilosula* under diverse concentrations of benzoic acid treatments. The upper portion showcases the phenotypic traits of potted plants, whereas the lower portion delineates the root morphology of *C. pilosula*.

Benzoic acid stress significantly inhibited the growth of *C. pilosula* (Figure 1). Under benzoic acid stress, the leaves of *C. pilosula* exhibited yellowing, gradually transitioning from green to yellow. Meanwhile, the development of the root system of *C. pilosula* was severely hindered; it is worth noting that the roots noticeably turned yellow and the formation of fibrous roots increased. The phenomenon of yellowing of leaves and proliferation of fibrous roots shows a clear quantitative relationship, which intensifies with increasing concentration of benzoic acid. As the concentration of benzoic acid gradually increased from 50 mmol/L to 300 mmol/L, we determined that the appropriate concentration for benzoic acid stress was 200 mmol/L.

**Figure 2:**
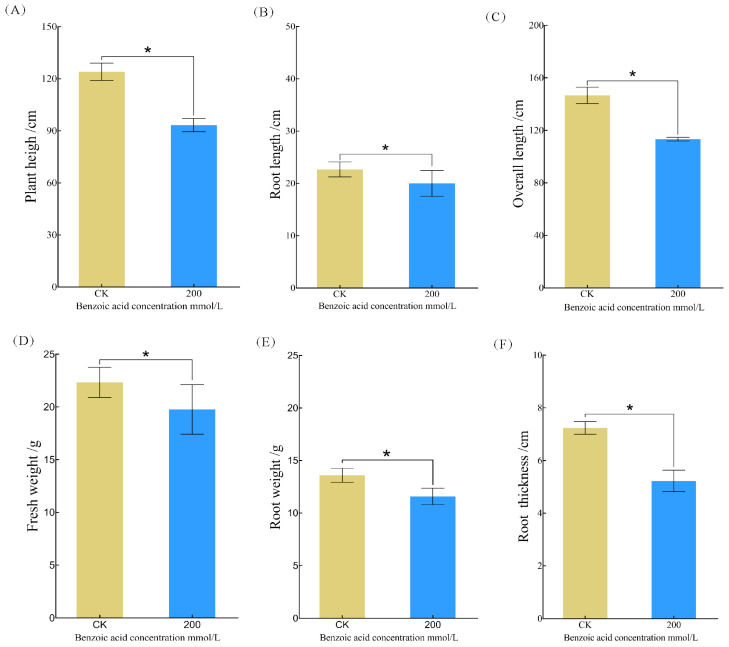
Morphological parametric assessments of *C. pilosula* upon exposure to benzoic acid at a concentration of 200 mmol/L: (**A**) Plant height; (**B**) root length; (**C**) total plant length; (**D**) fresh weight; (**E**) root weight; (**F**) root thickness. Note: “*” indicates significant differences between treatments at (*p* < 0.05).

The optimum concentration of 200 mmol/L for benzoic acid-challenged *C. pilosula* was ascertained on the basis of morphological parameters, succeeded by a meticulous determination of the same. The height (Figure 2A), root length (Figure 2B), total length (Figure 2C), fresh weight (Figure 2D), root weight (Figure 2E), and root thickness (Figure 2F) of *C. pilosula* were conspicuously attenuated by benzoic acid exposure, with decrements of 32.9%, 13.3%, 29.3%, 13.1%, 17.3%, and 38.5%, respectively (*p* < 0.05).

**Figure 3:**
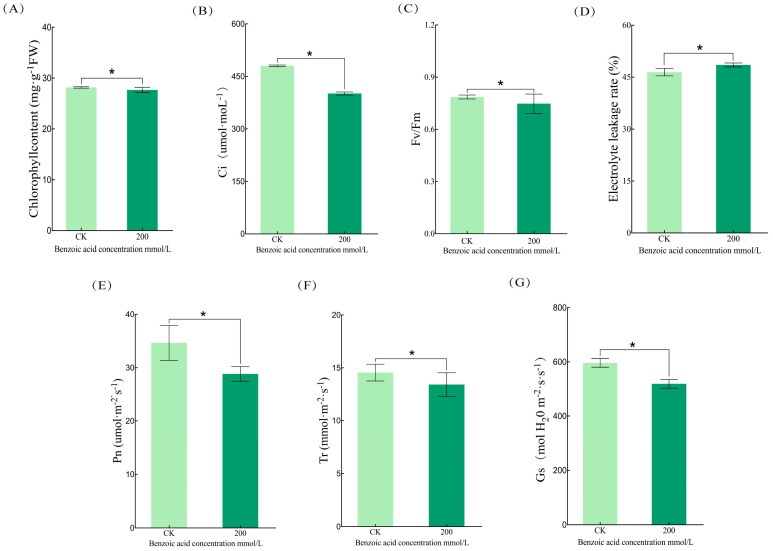
Photosynthetic parameters under varying concentrations of benzoic acid treatments: (**A**) chlorophyll content; (**B**) leaf electrolyte osmolality; (**C**) Fv/Fm ratio; (**D**) leaf intercellular CO_2_ concentration (Ci); (**E**) net photosynthetic rate (Pn); (**F**) leaf transpiration rate (Tr); (**G**) leaf stomatal conductance (Gs). Note: “*” indicates significant differences between treatments at (*p* < 0.05).

Benzoic acid conspicuously suppressed the photosynthetic performance of *C. pilosula*. Upon treatment with benzoic acid at a concentration of 200 mmol/L, the leaves manifested chlorosis, undergoing a gradual transition from green to yellow. The pertinent photosynthetic metrics were evaluated, disclosing that benzoic acid stress significantly curtailed the intercellular CO_2_ concentration (C_i_), chlorophyll fluorescence parameter (F_v_/F_m_), net photosynthetic rate (P_n_), transpiration rate (T_r_), and leaf stomatal conductance (G_s_) in the electric leaves of *C. pilosula* (Figure 3B,C,E–G) by 19.7%, 5.4%, 23.5%, 8.2%, and 14.8%, respectively; conversely, it elicited a 4.3% augmentation in leaf electrolyte osmolality. With the escalation of benzoic acid concentration, pronounced variations were witnessed in the photosynthetic efficiency, chlorophyll content, and leaf electrolyte osmolality of *C. pilosula* leaves.

**Figure 4:**
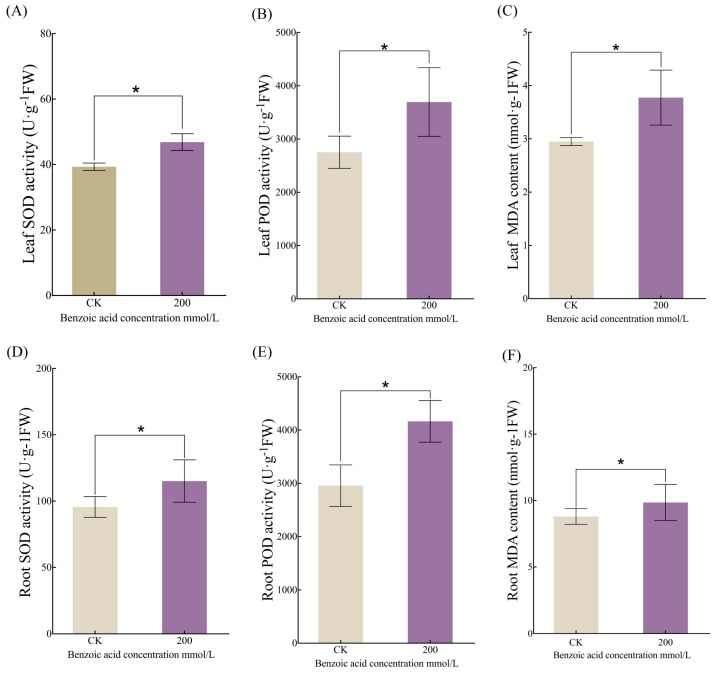
Physiological and photosynthetic metrics under diverse concentrations of benzoic acid administration: (**A**) leaf superoxide dismutase activity; (**B**) leaf peroxidase activity; (**C**) leaf malondialdehyde content; (**D**) root superoxide dismutase activity; (**E**) root peroxidase activity; (**F**) root malondialdehyde content. Note: “*” signifies statistically noteworthy discrepancies between treatments (*p* < 0.05).

Plants engage their antioxidant defense mechanisms to counteract deleterious external circumstances and sustain oxidative equilibrium when exposed to stressors. The current investigation revealed a substantial upsurge in the concentrations of superoxide dismutase (SOD), malondialdehyde (MDA), and peroxidase (POD) within both the foliar and root of *C. pilosula* under benzoic acid treatment at a concentration of 200 mmol/L (Figure 4A–F). Markedly, the levels of SOD, MDA, and POD were elevated in the roots in contrast to those in the leaves. Specifically, the activity of SOD in the leaves and roots of the benzoic acid-treated group was augmented by 16% and 17%, respectively (*p* < 0.05), compared to the control group (Figure 4A,D). Furthermore, the activity of POD increased by 25.5% and 28.9%, respectively; concurrently, the content of MDA escalated by 20.5% and 11.22%, all statistically significant at (*p* < 0.05).

**Figure 5:**
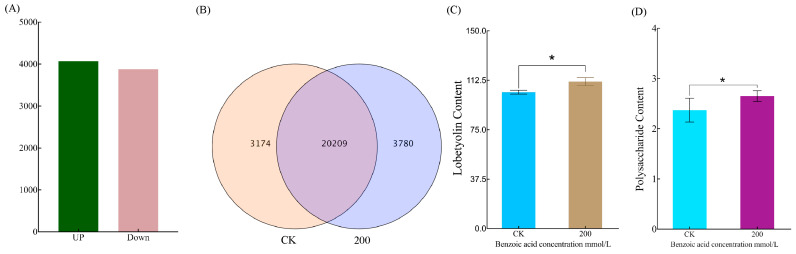
Expression profiling of genes associated with lobetyolin biosynthesis in the roots of *C. pilosula*: (**A**) Statistical charting of upregulated and downregulated genes in *C. pilosula* under control (CK) and benzoic acid (200 mmol/L) treatments; (**B**) shows a Venn plot depicting the cross expression of differentially expressed genes between CK and 200; Specifically, the two groups have a total of 20,209 genes, with 3174 and 3180 representing the genes uniquely expressed in each respective group; (**C**) fluctuations in lobetyolin content in the roots of *C. pilosula* under CK and benzoic acid (200 mmol/L) treatments; (**D**) modifications in polysaccharide content in the roots of *C. pilosula* under CK and benzoic acid (200 mmol/L) treatments. Note: “*” signifies statistically prominent disparities between treatments (*p* < 0.05).

In order to explore the mechanism of benzoic acid on the growth of *C. pilosula* root system, transcriptome sequencing was performed on the roots treated with 200 mmol/L benzoic acid. Analysis of eukaryotic reference transcriptomes (RNA-seq) from six samples revealed that the number of upregulated genes exceeded the number of downregulated genes (Figure 5A). Transcriptome sequencing of *C. pilosula* showed that there were 20,209 genes commonly expressed in both CK and 200 mmol/L benzoic acid treatment groups, while each group specifically expressed 3174 and 3180 genes respectively (Figure 5B). After treatment with 200 mmol/L benzoic acid, there was a significant increase in the content of *C. pilosula* lobetyol and polysaccharides compared to CK (Figure 5C,D).

**Figure 6:**
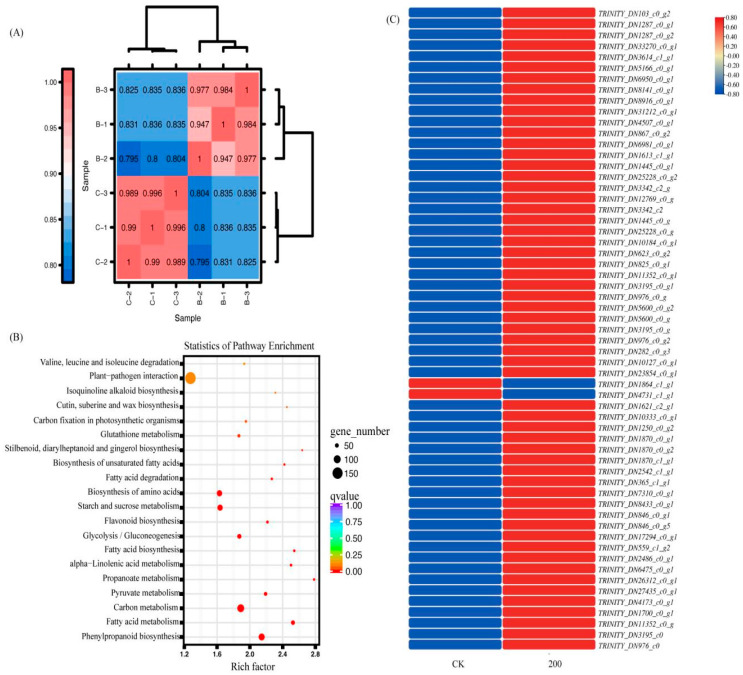
Heatmap illustrating sample expression correlation: (**A**) The magnitude of each color block represents the correlation between two samples along the x and y axes; the higher the magnitude, the stronger the correlation. (**B**) Bubble plot demonstrating KEGG enrichment analysis of differentially expressed genes in *C. pilosula* upon CK and benzoic acid (200 mmol/L) treatment: The size of the bubbles reflects the count of enriched genes, with larger bubbles indicating a greater abundance of enriched genes, and the bubble colors signify the enrichment significance—darker colors correspond to higher q-values, *p* ≤ 0.01 and fold change ≥ 1.2. (**C**) Distinctive expression profiles of genes encoding key enzymes in the kynurenine synthesis pathway in *C. pilosula* roots subjected to CK and benzoic acid (200 mmol/L) treatments.

Clustering analysis was performed on the treatment with 200mmol/L benzoic acid (Figure 6A), revealing a complete match between the sample and the detected results. KEGG enrichment analysis was conducted on this treatment (Figure 6B), identifying pathways such as valine, leucine, and isoleucine degradation, plant-pathogen interaction, isoquinoline alkaloid biosynthesis, cutin, suberine and wax biosynthesis, carbon fixation in photosynthetic organisms, glutathione metabolism, stilbenoid, diarylheptanoid and gingerol biosynthesis, biosynthesis of unsaturated fatty acids, fatty acid degradation, biosynthesis of amino acids, starch, sugar metabolism, flavonoids, glycolysis/gluconeogenesis, fatty acid biosynthesis, alpha-linolenic acid metabolism, propanoate metabolism, pyruvate metabolism, carbon metabolism, fatty acid metabolism, and phenylpropaniod biosynthesis. The corresponding differentially expressed genes (DEGs) within these pathways were analyzed using normalized gene expression values calculated from FPKM counts transformed by TBtools (2.0). The red color indicates upregulation, while the blue color represents downregulation (Figure 6C).

**Figure 7:**
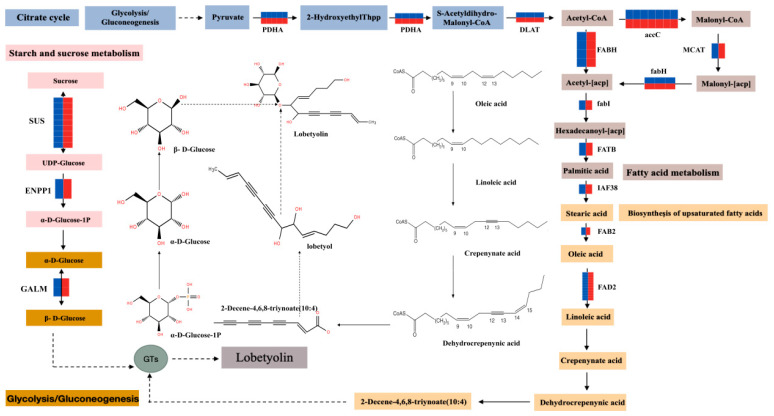
Schematic illustration of the biosynthetic route for lobetyolin in *C. pilosula.* Dotted arrowhead indicates an indirect effect.

The biosynthesis of lobetyolin in *C. pilosula* traverses the citric acid cycle, glycolysis, and gluconeogenesis pathways. Acetoacetate undergoes conversion to 2-Hydroxyethylthioester (2-HydroxyethylThpp) by the enzyme pyruvate dehydrogenase complex (PDC). Subsequently, 2-Hydroxyethylthioester is transmuted into S-Acetyldihydromalonyl-CoA through the action of PDC, which is then transformed into acetyl-CoA by the enzyme fatty acyl-ACP thioesterase B (FABH), signifying the inception point of fatty acid synthesis. Acetyl-CoA forms an acetyl carrier (Acetyl-ACP) in conjunction with 3-oxoacyl-[acyl carrier protein] synthase II under the catalysis of FABH, and undergoes a succession of enzymatic reactions to engender a palmitoylcarrier (Hexadecanoyl-[ACP]). Under the impetus of Fatty acyl ACP thiosterase B (FATB), this palmitoyl carrier is successively converted into saturated fatty acid palmitic acid. Palmitic acid is further transfigured into stearic acid via an unsaturated fatty acid pathway catalyzed by Very Long Chain 3-Oxoacyl-CoA Reductase (IAF38), followed by its conversion to oleic acid under the regulation of acid-[acyl carrier protein] desaturase (FAB2). Oleic acid subsequently gives rise to linoleic acid under the influence of omega-6 fatty acid desaturase (FAD2). Linoleic acid can be metamorphosed to crepenynate by the action of Δ9 desaturase; moreover, Δ12 desaturase may introduce a new double bond at the Δ9 position of crepenynate, resulting in dehydrocupenynate. Dehydrocupenynate can be further processed into 2-Decene-4,6,8-triynoate (10:4) through pathways potentially encompassing β-Oxidation taking place within the mitochondria or peroxisomes of cells that eliminate carbon atoms from fatty acid chains through multiple cycles while generating acetyl-CoA. In the metabolic routes of starch and sucrose, sucrose undergoes conversion from sucrose synthase (SUS) to UDP-glucose, and is then transformed into α-D-Glucose-1P by external nucleotide pyrophosphatase (ENPP1). α-D-Glucose-1P participates in both glycolytic and gluconeogenic cycles, leading to its conversion into α-D-glucose which is subsequently converted to β-D-glucose facilitated by aldose 1-isomerase (GALM), furnishing precursors indispensable for the synthesis of lobetyolin in *C. pilosula*. Ultimately, glucose moieties are glycosidically linked with lobetyol alcohol under the catalytic effect of glycosyltransferases (GTs) to form *C. pilosula* lobetyol (Figure 7). Additionally, the results evidenced that the expression levels, quantified as FPKM values, of key regulatory enzyme-coding genes within these pathways were conspicuously upregulated under the stress imposed by 200 mmol/L benzoic acid treatment. Consequently, this study postulates that the biosynthesis of lobetyolinin *C. pilosula* is intimately associated with the differential expression patterns of key enzyme genes involved in the citric acid cycle metabolism as well as those governing fatty acid and starch/sucrose metabolism.

**Figure 8:**
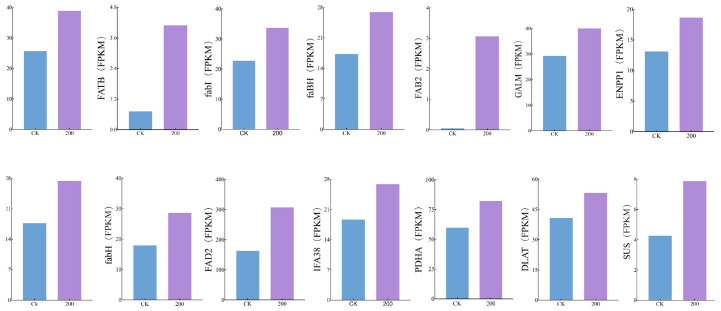
FPKM trend profiling of enzyme-encoding genes relevant to the biosynthetic trajectory of lobetyolin in *C. pilosula*.

The FPKM magnitudes of enzyme-encoding genes pertinent to the biosynthetic itinerary of lobetyolin in *C. pilosula* manifested pronounced upregulation (Figure 8).

**Figure 9:**
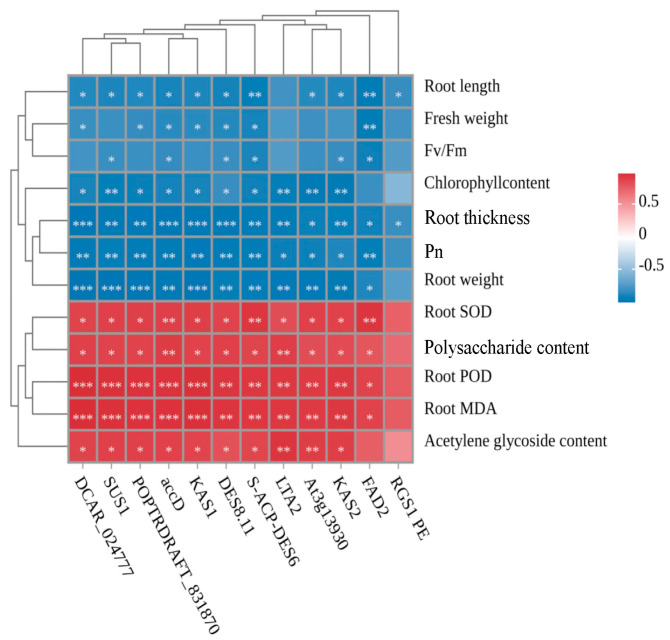
Dynamic correlation heatmap annotation. Note: “*” designates significant disparities between treatments (*p* < 0.05); “**” and “***” signify highly significant variances between treatments, (*p* < 0.01) and (*p* < 0.001) respectively.

**Figure 10:**
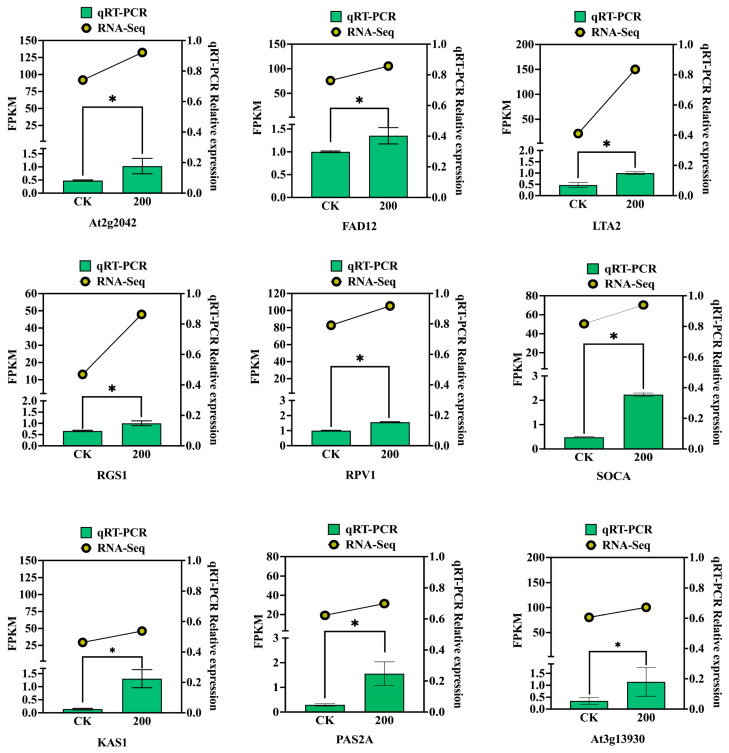
qRT-PCR analysis was conducted to examine the changes in DEGs in *C. pilosula* roots under CK and 200 mmol/L benzoic acid treatment. Nine DEGs that regulate key metabolic pathways were selected for qRT-PCR validation. The qRT-PCR values were compared with gene FPKM values to verify the reliability of transcriptomic data. “*” represents the results of Duncan’s multiple range test, indicating statistically significant differences (*p* < 0.05).

The root length, fresh weight, Fv/Fm ratio, chlorophyll content, and root thickness of *C. pilosula* were all adversely affected by treatment with benzoic acid (200 mmol/L), under the modulation of enzymes (SUS1, DCAR, At3g13930, KAS1, FAD2, DES8.11, accD, KAS2 S-ACP-DES6 LTA2 POPTRDRAFT-831870). However, in contrast to this negative effect on growth parameters mentioned above, the concentrations of reactive oxygen species scavenging enzymes (SOD and POD), malondialdehyde (MDA) content, as well as polysaccharides and alkynes were positively modulated by these enzymes. This implies that enzymatic activity, polysaccharide accumulation, and lobetyolin biosynthesis in the roots of *C. pilosula* are augmented via the aforementioned genes under the stress of benzoic acid. Consequently, it indicates that lobetyolin might act as a potential metabolite for *C. pilosula* to counteract benzoic acid-induced stress (Figure 9). To verify the reliability of transcriptome data, this study randomly selected nine genes for qRT-PCR validation. The results showed that the qRT-PCR data was consistent with the gene expression levels in the transcriptome, indicating the accuracy of the transcriptome data (Figure 10).

## 3. Discussion

Benzoic acid, as a stress factor, has had a significant impact on plant growth and development. This study shows that benzoic acid has a significant inhibitory effect on the growth and development of *Polygonatum*, and it can significantly affect the growth rate of watermelon root systems. As the concentration of benzoic acid increases, abnormalities appear in the nucleus of the root tip cells, in the nucleolus, sparse cytoplasm, and enlarged and increased vacuoles [25]. Moreover, this study disclosed that 200 mmol/L benzoic acid provoked severe leaf chlorosis of *C. pilosula*, an augmentation in fibrous roots, and a curtailment of main root length. These revelations imply that the accumulation of allelopathic substances such as benzoic acid within the root system might constitute one of the predominant factors hampering the growth of *C. pilosula* plants and roots under continuous cropping circumstances. Benzoic acid stress elicited marked alterations in the photosynthetic efficacy, chlorophyll content, and leaf electrolyte permeability of *C. pilosula* leaves. These findings are in agreement with previous studies on poplar trees [26]. Additionally, benzoic acid stress augmented the antioxidant enzyme activity in both leaves and roots of *C. pilosula*. Under the treatment of a benzoic acid concentration of 200 mmol/L, there was a remarkable escalation in the content of superoxide dismutase (SOD), malondialdehyde (MDA), and peroxidase (POD) in both leaves and roots (Figure 4A–F), with the content of SOD, MDA, and POD in the roots being higher than that in the leaves. When plants are exposed to excessive benzoic acid, a copious amount of reactive oxygen species (ROS) is generated [27]. Higher production of ROS can give rise to oxidative stress [28,29]. The results of this study suggest that substantial variations occur in the morphology, photosynthetic parameters, aboveground enzymatic activity, and underground enzymatic activity of *C. pilosula* plants under benzoic acid stress.

Benzoic acid might indirectly impinge upon the metabolism of starch and sucrose by manipulating the structural integrity and functional dynamics of plant cells [30]. Benzoic acid stress exerts a profound influence on the formation and sugar metabolism of potato in vitro tubers, particularly with regard to the content of sucrose and starch, and the related enzymatic activities, signifying that such stress can modify both the accumulation and metabolic routes of carbohydrate substances in plants [31]. The synthesis of sucrose-6-benzoate requires specific catalysts and reaction conditions; optimizing these parameters can facilitate the enhancement of both yield and product quality [32]. Under benzoic acid stress, physiological and biochemical attributes within plants undergo remarkable alterations. For instance, the embryonic roots of cucumber seeds display variations in amylase activity along with alterations in the antioxidant enzyme system activity under this stress condition. These modifications might indirectly affect the biosynthetic pathways of starch and sucrose [33]. Studies on banana fruits suggest a significant positive correlation between the formation of resistant starch and the total content of starch and sucrose [34], indicating a similar positive interrelationship between starch and sucrose levels under benzoic acid stress [35]. This study disclosed that key enzyme-encoding genes implicated in the metabolic pathways of starch and sucrose were upregulated within the root system of *C. pilosula* under benzoic acid stress, potentially constituting a primary factor contributing to the augmentation of lobetyolin content. Benzoic acid may indirectly impinge upon the tricarboxylic acid (TCA) cycle by manipulating the equilibrium of carbon and nitrogen metabolism in plants. For example, benzoic acid might exert an inhibitory effect on the overall efficiency of the TCA cycle by suppressing the enzymatic activity or modulating the expression profiles of specific TCA cycle enzymes. In response to stress, plants may potentiate the activity of certain metabolic pathways to attenuate adverse impacts or optimize energy utilization and biosynthesis by regulating crucial nodes within the TCA cycle [36,37]. Research findings suggest that a multitude of plant species orchestrate their organic acid metabolism in response to environmental stressors. Citric acid, as a low-molecular-weight organic acid, assumes a crucial role in diverse physiological metabolic processes both intracellularly and extracellularly. Hence, under benzoic acid stress conditions, plants may recalibrate the metabolic trajectory of citric acid to accommodate adverse circumstances. Benzoic acid treatment has been demonstrated to significantly augment the activities of peroxidase (POD) and superoxide dismutase (SOD) in fruits, indicating that under such stress conditions, plants may respond by enhancing the citric acid metabolic pathway [38]. Isocitrate dehydrogenase (ICDH), a key enzyme bridging carbon-nitrogen metabolism, catalyzes the reversible oxidative decarboxylation of isocitrate to form α-ketoglutarate within the TCA cycle [39]. Under benzoic acid stress, the activity of ICDH may be compromised, thereby exerting an impact on the overall efficiency of the TCA cycle [40]. This effect might be correlated with the production and consumption of POD in plants, influencing nearly all metabolic pathways [41]. Additionally, benzoic acid stress may adversely affect the functionality of plant roots, thereby indirectly influencing the citric acid cycle [42]. The root system serves as the primary organ for nutrient and water uptake; its dysfunction may impede the effective utilization of citric acid for energy generation and biosynthesis [43,44,45]. In the context of the benzoic acid concentration stress in this experiment, key genes implicated in the citric acid cycle (*Din019274*, *DCAR024777*, *SCOAAt2g20420*) were upregulated, potentially influencing the accumulation of lobetyolin.

Benzoic acid, as an autotoxic compound, elicits diverse magnitudes of impact on protective enzymes such as superoxide dismutase (SOD) and peroxidase (POD) in Chinese fir seedlings experiencing nutritional stress [46]. Amidst conditions of nutrient deficiency—specifically calcium or phosphorus deficiency—low concentrations of benzoic acid initially induce a decline followed by an increase in the activity of these protective enzymes, while higher concentrations engender an overall attenuation in enzymatic activity [47,48]. This implies that benzoic acid might orchestrate plant responses to stress by influencing the antioxidant system within a definite concentration range, which could potentially have an indirect bearing on fatty acid metabolism. From the standpoint of fatty acid desaturases, they play a pivotal role in mediating plant responses to both abiotic and biotic stressors [49,50]. Consequently, benzoic acid stress could potentially modify the activity or expression profiles of fatty acid desaturases, thereby influencing the desaturation process, mobilization, and regulation of fatty acids, and ultimately affecting plant stress tolerance. In this study, benzoic acid stress led to an upsurge in the expression levels of genes encoding fatty-acid-related synthases (IFA38, FAD2), further signifying that benzoic acid stress may regulate the physiological status of plants by modulating key enzymes implicated in the fatty acid metabolic pathways to accommodate adverse environmental circumstances.

*C. pilosula* lobetyolin, categorized as a polyethylene block glycoside, is proffered as an indicator compound for *C. pilosula* lobetyolin and serves as a potential biomarker associated with hematopoietic and immune functions [51]. Moreover, it plays a vital role in the anti-tumor effect against lung cancer. Hence, exploring the biosynthetic process of *C. pilosula* lobetyolin assumes paramount significance [52]. The synthesis of this compound might emanate from the metabolic routes encompassing sucrose and starch. Initially, sucrose is transmuted to UDP-glucose under the catalysis of sucrose synthase (SUS) [53]. Subsequently, via the action of external nucleotide pyrophosphatase (ENPP1), UDP-glucose is further transformed into α-D-glucose-1-phosphate [54]. This intermediate entity participates in the glycolytic and gluconeogenic cycles before being ultimately converted to α-D-glucose [55]. Under the catalysis of aldose 1-isomerase (GALM), α-D-glucose is reconverted to β-D-glucose [56]. Acetoacetate undergoes conversion to acetyl-CoA through the glycolytic and gluconeogenic pathways, which serves as the incipient point for fatty acid biosynthesis [57]. Acetyl-CoA subsequently complexes with acyl carrier protein (ACP) to form an acetyl carrier protein (Acetyl-ACP), which then proceeds through a series of enzymatic reactions to yield the palmitoyl carrier (Hexadecanoyl-[ACP]) [58]. Catalyzed by the FATB enzyme, the palmitoyl support is further converted into saturated fatty acid palmitic acid [59]. Palmitic acid is converted to stearic acid via the unsaturated fatty acid pathways and is further transformed into oleic acid under the regulation of IFA38. Oleic acid is further converted to linoleic acid under the action of the FAD2 enzyme [60]. Linoleic acid is acted upon by the desaturase Δ9 to generate crepenynate acid, while the Δ12 desaturase may introduce a new double bond at the Δ9 position of crepenynate acid to form dehydrocrepenynate acid [61]. Eventually, dehydrocrepenynate acid might be converted to 2-Decene-4,6,8-triynoate through a pathway involving β-oxidation. This compound might then undergo successive hydrogenation, oxidation, and reduction reactions to form *C. pilosula* lobetyolin, potentially furnishing precursor substances necessary for the synthesis of *C. pilosula* lobetyolin.

The synthetic precursors of *C. pilosula* lobetyolin glycosides comprise glycosides and lobetyol [62], with glycosyltransferase (GT) potentially serving as a pivotal enzyme in the reaction between these two components. Glycosyltransferases exhibit multifaceted functions, wherein glycosylation is fundamental to the synthesis of complex and structurally diverse glycosides, thereby enhancing the biological activity and bioavailability of glycosidic ligands [63,64]. Triterpenoid saponins represent natural products characterized by diverse structures and distinct skeletons, and are modified by multiple sugar moieties [65,66]. In plants, the glycosylation of triterpenoid saponins can be catalyzed by GT; for instance, GT in *C. pilosula* plays a crucial role in generating various ginsenosides [67,68]. The process of triterpene glycosylation typically involves polysaccharide modifications that include branched glycosylation following primary reactions [69]. *C. pilosula* lobetyol alcohol reacts with β-D-glucose under the influence of GT to yield *C. pilosula* lobetyolin. The biosynthesis of both glycosides and unsaturated fatty acid chains is intricately linked to starch and sucrose metabolism, glycolysis/gluconeogenesis pathways, citric acid cycle dynamics, fatty acid metabolism processes, unsaturated fatty acid biosynthesis mechanisms, and linoleic acid metabolism [70]. This study compared expression levels (FPKM values) of key enzyme genes in roots of *C. pilosula* treated with 200 mmol/L benzoic acid. It was observed that genes encoding enzymes related to glycoside synthesis (such as SUS, ENPP1, and GALM) were upregulated alongside an increase in polysaccharide content from *C. pilosula* under continuous cropping conditions—consistent with measured polysaccharide levels. Under benzoic acid treatment conditions, genes associated with fatty acid chain synthesis (such as IF38 and FAB2) were also upregulated while those involved in unsaturated fatty acid (linoleic acid) metabolism (such as FAD2) exhibited increased expression; this may have contributed to elevated levels of *C. pilosula* lobetyolin. These findings align with HPLC detection results, indicating that exposure to a concentration of 200 mmol/L benzoic acid stress influences gene expression patterns, which may enhance the synthesis pathway for *C. pilosula* lobetyolin.

## 4. Materials and Methods

### 4.1. Materials

The test variety of *Codonopsis pilosula* (Franch.) Nannf. utilized in this study was ‘White Striped Codonopsis’ (*Codonopsis pilosula* (Franch.) Nannf.), sourced from Gansu Province, with an age of one year. The specifications for the seedlings included a length of 10–15 cm and a diameter ranging from 2 to 3 cm, ensuring uniformity in size among specimens. Seedlings were cultivated in plastic pots measuring 20 cm × 19 cm, filled with nutrient-rich soil. Three plants were planted per pot, and maintained at a temperature of 23 °C. Throughout the growth period, all specimens received equal watering treatment. After a growth duration of 45 days (seedling stage), aboveground materials (stems and leaves) as well as belowground components (roots) were thoroughly rinsed with distilled water and subsequently stored separately at −80 °C.

In this study, rhizosphere soil samples (fresh) of *C. pilosula* were collected in August 2023 from the large-scale experimental base for continuous cropping systems of *C. pilosula* located in Lintao County, Dingxi City, Gansu Province, China (longitude 103°29′08′′–104°19′34′′ E; latitude 35°03′42′′–35°56′46′′ N) for the extraction of chemosensitive substances. The collection treatments included rhizosphere soil samples from fields with positive crop growth and those subjected to continuous planting for durations of 1, 2, 3, and 4 years. Five sampling points were randomly selected within each treatment group and combined into a single sample for three biological replicates. The collected soils were subsequently encapsulated and stored at a temperature of 4 °C in a refrigerator until required for experimental analysis.

### 4.2. Experimental Methods

#### 4.2.1. Extraction of Chemosensitive Compounds

A total of 25 g of rhizosphere soil from *C. pilosula* (fresh sample) was ground and passed through a 40-mesh sieve into a 200 mL triangular flask. Subsequently, 100 mL of 80% methanol was added, and the mixture was subjected to ultrasonic extraction for 3 h. The resulting solution was centrifuged, and the supernatant was collected and rotary evaporated to yield an aqueous phase volume of approximately 20 mL. The pH of this solution was adjusted to 3.0 using 1 mol/L HCl, followed by three successive extractions with ethyl acetate. The ethyl acetate phase obtained from these extractions underwent further treatment with 20 mL of 8% NaOH in three separate rounds. The resultant aqueous phase was extracted again using 1 mol/L HCl at a temperature of 45 °C, after which the ethyl acetate phase acquired via rotary evaporation underwent three additional extractions. Finally, the aqueous phase derived from extraction had its pH adjusted back to 3.0 with hydrochloric acid before being extracted thrice with an equal volume (20 mL) of ethylacetate; this ethyl acetate extract was then concentrated to dryness via rotary evaporation at a temperature of 45 °C and subsequently re-dissolved in ethyl acetate to achieve a final volume of 1 mL for preservation in a micropore membrane filter (0.45 μm) for future use.

#### 4.2.2. Silanization Procedure of Rhizosphere Soil Samples

The aforementioned concentrated specimens underwent silanization with a BSTFA:pyridine (5:1) mixture, upon addition of 0.25 mL of the silanizing agent. Subsequently, the specimens were capped, hermetically sealed, and underwent derivatization in a water bath maintained at 80 °C for a period of 2 h prior to being prepared for measurement.

#### 4.2.3. Gas Chromatography-Mass Spectrometry Profiling of Chemosensory Entities

An Agilent 7890A–7000B mass spectrometric system (Agilent Technologies, Santa Clara, CA, USA) was utilized for analytical determination. The electron bombardment source was operated at a voltage of 70 eV, with a scanning range of *m*/*z* 30 to 600 atomic mass units (AMU) and a scanning velocity of 0.2 s per sweep throughout the entire procedure; the ion source temperature was maintained at 230 °C. The chromatographic column employed was DB-5MS (5 m × 0.25 mm × 0.5 μm), featuring a stationary phase constituted of 5% phenyl-methyl polysiloxane. The initial temperature was set at 50 °C and held for 2 min before being programmed to ascend to 250 °C at a rate of 6 °C/min, where it persisted for an additional 15 min. The inlet port temperature was maintained at 250 °C, with high-purity helium serving as the carrier gas at a flow rate of 1 mL/min; the injection volume was stipulated accordingly. Mass spectral data were analyzed by recourse to the NIST08 (chemdata.nist.gov, accessed on 6 November 2023) mass spectrometry database to ascertain the identities of the components.

#### 4.2.4. Evaluation of Growth Metrics

The vine length, root length, and root diameter of the tagged *C. pilosula* specimens were respectively gauged with a tape measure, a ruler, and a vernier caliper. The roots were then cleansed to eliminate adherent water droplets and desiccated with absorbent paper. The fresh masses of both the roots and the above-ground portions were ascertained using a high-precision balance with a resolution of one-thousandth of a gram.

#### 4.2.5. Quantification of Photosynthetic Attributes

The LI-340 portable photosynthesizer (Li-Cor, Lincoln, NE, USA) was utilized for the determination and quantification of photosynthetic parameters within the time frame of 7:00 to 9:00 a.m. The evaluated photosynthetic attributes encompassed net photosynthetic rate (Pn), stomatal conductance (Gs), intercellular CO_2_ concentration (Ci), and transpiration rate (Tr).

#### 4.2.6. Evaluation of Chlorophyll Fluorescence Indices

Prior to measurement, the plants were acclimated in a dark environment for 30 min, after which chlorophyll fluorescence parameters were assessed using a portable chlorophyll fluorometer (FluorPen FP 100, Ecotech, Beijing, China). Key metrics such as the ratio of maximum photochemical efficiency to initial fluorescence (Fv/Fm) and non-photochemical quenching (qN) were calculated utilizing the integrated data processing software PAM Win (FluorPen 1.0). The leaves employed for determining chlorophyll fluorescence parameters were identical to those used for assessing photosynthetic characteristics.

#### 4.2.7. Evaluation of Antioxidant Enzyme Potencies

SOD and POD activities were gauged in accordance with the assay methodology delineated by [71]. A 0.25 g sample was positioned in a pre-chilled mortar, and 2 mL of the extraction medium (phosphate-buffered saline/trichloroacetic acid [TCA]) was incorporated. The concoction was macerated to generate a homogenous suspension and transferred to a centrifuge tube, with an extra 3 mL of the extraction medium used for rinsing the mortar to attain a total volume of 5 mL. The resultant mixture was centrifuged at 10,000× *g* for 10 min at 4 °C to obtain the crude enzyme extract for subsequent quantification of enzyme activity.

#### 4.2.8. Estimation of Malondialdehyde Content

Malondialdehyde (MDA) content was gauged in consonance with the assay methodology delineated by [71]. A 0.25 g sample was positioned in a pre-chilled mortar, and 2 mL of the extraction medium (thiobarbituric acid [TBA]) was incorporated. The concoction was macerated to engender a homogeneous emulsion and transferred to a centrifuge tube, with an extra 3 mL of the extraction medium employed for rinsing the mortar to attain a cumulative volume of 5 mL. The crude enzyme extract for subsequent quantification of enzyme activity was procured by centrifugation at 10,000× *g* for 10 min at 4 °C. Absorbance was gauged at 600 nm utilizing an enzymatic marker, and MDA content was computed.

#### 4.2.9. Evaluation of Relative Electrical Conductivity (REC)

Relative electrical conductivity (REC) was appraised in line with the determination approach postulated by [72]. At the outset, the leaves were scrupulously rinsed thrice with deionized water, and superfluous moisture was expunged from their surfaces via clean filter paper. Subsequently, 0.3 g of excised leaves were lodged in a 50 mL centrifuge tube encompassing 30 mL of deionized water (designated as EL1). An extra centrifuge tube containing 30 mL of deionized water functioned as a control (designated as EL0). The tubes housing the leaf specimens were then stationed on a shaker (QB-206, Qilinbel Instrument Manufacturing Co., Ltd., Haimen, China) at ambient temperature; conductivity readings for both the samples (EL1) and the controls (EL0) were procured after one hour of oscillation at 20 rpm. After this measurement, the tubes were boiled for 10 min and then permitted to cool to ambient temperature. Conductivity estimations for both the sample (denoted as EL3) and the blank control (denoted as EL2) were acquired anew using a DDS-307 conductivity meter (Remco, Shanghai, China), adhering to the formula: EL (%) = [(EL1 − EL0)/(EL3 − EL2)] × 100%.

#### 4.2.10. Evaluation of Lobetyolin Content in *C. pilosula*

The assessment of lobetyolin in *C. pilosula* was executed in adherence to the protocol stipulated by [73]. The content of lobetyolin was ascertained via high-performance liquid chromatography (HPLC), and the chromatographic parameters were as follows: an Agilent Extend-C18 column (5 µm, 250 mm × 4.6 mm, Agilent Technologies, Santa Clara, CA, USA) was employed; the mobile phase constituted a mixture of acetonitrile and water at a ratio of 20:80; the flow rate was calibrated at 1 mL/min; the detection wavelength was set to 267 nm; the column temperature was maintained at 25 °C; and the injection volume was fixed at 10 µL.

#### 4.2.11. Evaluation of Polysaccharide Content in *C. pilosula*

The methodology for the quantification of polysaccharides in *C. pilosula* was adapted from the assay developed by [74]. A dried sample weighing 0.2 g of *C. pilosula* was finely ground into a powder and subsequently treated with 80% ethanol under ultrasonic conditions for 2 h. The mixture was then filtered to discard the filtrate, after which the residue was transferred to a conical flask and treated with 80 mL of boiled distilled water. Ultrasonic extraction was performed for an additional 30 min before filtering again. Subsequently, 5 mL of the resulting filtrate was diluted with 45 mL of distilled water; this solution underwent further treatment by adding 1 mL to a test tube along with 1 mL of phenol and then introducing 5 mL of concentrated sulfuric acid. The mixture was boiled for 10 min, followed by measurement of absorbance at a wavelength of 279 nm.

#### 4.2.12. Comprehensive RNA Extraction, Library Establishment, and Transcriptome Sequencing

The roots of *C. pilosula* exposed to diverse treatments and fully frozen were pulverized in liquid nitrogen, and total RNA was extracted from the root tissues by employing a TRIzol^®^ (Tiangen, Beijing, China) reagent. The purity and concentration of the extracted RNA were appraised through a NanoDrop 2000 spectrophotometer (Thermo Fisher Scientific, Waltham, MA, USA), while the integrity of the RNA was scrupulously examined using an Agilent 2100 Bioanalyzer/LabChip GX (Agilent Technologies, Santa Clara, CA, USA). Once the samples passed the quality checks, library construction was initiated. Eukaryotic mRNA was enriched with magnetic beads conjugated to Oligo(dT); mRNA fragmentation was accomplished by adding Fragmentation Buffer; the synthesis of the first cDNA strand and double-stranded cDNA was conducted using mRNA as a template, followed by purification processes for the generated cDNA. The purified double-stranded cDNA underwent end repair, addition of A-tails, and ligation of sequencing adapters prior to sequencing via AMP technology. Fragment size selection for optimization was carried out using AMPure XP beads (Beckman Coulter, Brea, CA, USA); ultimately, PCR amplification augmented the constructed cDNA library. After library construction, initial quantification was performed with a Qubit 3.0 Fluorometer to ensure the concentration reached at least 1 ng/µL or higher. Subsequently, the insertion fragments within the library were analyzed using a Qsep400 High-Throughput Analysis System (BiOptic Inc., New Taipei City, Taiwan); upon confirmation of meeting expectations, quantitative PCR (Q-PCR) precisely determined the effective library concentration (>2 nM) to guarantee that the quality benchmarks were satisfied. Index-coded samples were clustered on the cBot Cluster Generation System through the utilization of TruSeq PE Cluster Kit v3-cBot-HS (Illumina, San Diego, CA, USA). After cluster generation completion, sequencing preparations were executed on the designated platform, generating paired-end reads of 150 bp.

#### 4.2.13. Data Processing, Transcriptome Assembly and Functional Annotation

We implemented stringent data quality control measures, including the removal of reads containing splicing fragments and low-quality sequences, resulting in the acquisition of high-quality clean data. Subsequently, Trinity software (2.14.0) [75] was employed to generate a comprehensive set of fragments from the obtained high-quality sequencing data. Finally, we utilized De Bruijn graph analysis and sequencing read information to identify transcriptional sequences within each fragment set separately. The assembly results were evaluated based on the N50 value as a measure of quality. Gene expression levels were estimated using RSEM (v1.2.19)[76] through Fragments Per Kilobase Million (FPKM) calculations. Differential gene expression analysis for RNA sequencing data was performed using the DEGseq algorithm (1.39.0), with parameters set at *p* ≤ 0.01 and fold change ≥ 1.2 [77]. Functional annotation was conducted utilizing various databases, including GO (Gene Ontology), KOG (EuKaryotic Orthologous Groups), and KEGG [78] (Kyoto Encyclopedia of Genes and Genomes).

#### 4.2.14. Quantitative Real-Time PCR

To verify the reliability of the transcriptome sequencing data, nine DEGs were selected from the transcriptome data for qRT-PCR. Three replications were made for each treatment. The qRT PCR primer sequence information can be found in Supplementary Table S1. qRT-PCR was performed after RNA reverse transcription using a LightCycler^®^96 Real time-PCR machine (Roche, Mannheim, Germany). The reaction system used a total of 20 µL: 10 µL of 2× Talent qPCR premix, 6.8 µL of RNase-Free ddH_2_O, 2 µL of 75 ng/µL cDNA, and the forward and reverse primers of 10 µmol·L^−1^ were 0.6 µL each. The qRT-PCR reaction program was used as referenced by Lu et al. [79]. The relative expression of nine genes was calculated by the 2^−∆∆Ct^ method, using β-tubulin [80] as the housekeeping gene.

#### 4.2.15. Data Manipulation and Elaboration

All experiments were executed in a fully randomized design and replicated thrice. The analysis of variance (ANOVA) was accomplished via GraphPad Prism software (version 9.0), whilst SigmaPlot 12.5 was enlisted for graphical delineation.

## 5. Conclusions

In sum, lobetyolin functions as a pivotal indicator for appraising the quality of *C. pilosula* and constitutes one of the predominant active constituents extracted from its root system. Consequently, disclosing the macro- and micro-mechanisms governing root growth and lobetyolin accumulation in response to benzoic acid stress is indispensable for fostering a high-caliber industry centered on *C. pilosula*. In this research, it was discerned that treatment with 200 mmol/L benzoic acid hindered the growth and development of *C. pilosula*, giving rise to conspicuous morphological variations. Moreover, the photosynthetic activities and antioxidant enzyme systems of *C. pilosula* were substantially compromised by benzoic acid. The biosynthesis of lobetyolin in *C. pilosula* was intimately correlated with the differential expression of key enzymatic genes implicated in the citric acid cycle, fatty acid metabolism, and starch and sucrose metabolism pathways—processes that are intrinsically associated with the plant’s resistance to abiotic stresses. Notably, the expression magnitudes of up-regulated genes surpassed those of down-regulated genes within these metabolic contexts, intimating that 200 mmol/L benzoic acid stress exerted an accumulative influence on lobetyolin production in *C. pilosula*. This revelation further implies that such stress might augment the lobetyolin content within *C. pilosula*, suggesting its potential as a defense substance against abiotic stresses.

## Figures and Tables

**Table 1:** Types of soil chemical susceptibility substances.

Chemical Name	Molecular Formula	Molecular Mass
Octamethylcyclotrisiloxane	C_8_H_24_O_2_Si_3_	236.53
Hexamethyldisiloxane	C_6_H_18_OSi_2_	162.37
Trifluoroacetamide	C_2_H_2_F_3_NO	133.04
Diethylamine	C_4_H_11_N	73.137
Ethylamine	C_2_H_7_N	45.084
N,N-Diethyl(trimethylsilyl)carb amate	C_8_H_19_NO_2_Si	189.022
Glycine, N-(trimethylsilyl)-, trimethylsilyl ester	C_8_H_21_NO_2_Si_2_	219.063
Ethylene glycol	(CH_2_OH)_2_	62.068
Acetamide	C_2_H_5_NO	59.067
Crotonic acid	C_4_H_6_O_2_	86.089
Octyl caprylate	C_16_H_32_O_2_	256.424
Tris(trimethylsilyl) phosphate	C_9_H_27_O_4_PSi_3_	314.5
Benzoic acid	C_7_H_6_O_2_	122.1214
3-Chloro-1,2-propanediol	C_3_H_7_ClO_2_	110.539
Ferulic acid	C_10_H_10_O_4_	194.184
2,6-Di-tert-butylphenol	C_14_H_22_O	206.33
Acetamide	C_2_H_5_NO	59.067

## Data Availability

The original contributions presented in the study are included in the article, further inquiries can be directed to the corresponding authors.

## Supplementary Materials

The following supporting information can be downloaded at: https://www.mdpi.com/article/10.3390/ijms252011007/s1.
