# Supplementary material for: Research on the Mechanism of Growth of Codonopsis pilosula (Franch.) Nannf. Root Responding to Phenolic Stress Induced by Benzoic Acid
Source: Int J Mol Sci. 2024 Oct 13;25(20):11007. doi: 10.3390/ijms252011007 (PMC11508040; doi:10.3390/ijms252011007)
Supplement: Supplementary file 1 [file ijms-25-11007-s001.zip › ijms-3235342-supplementary.pdf]

Supplementary Table

Table S1. Primer sequences.

Gene name	Forward primer	Reverse primer
PP2A	GGCTTACTTCCCTCTCCTTGG	TATCCCCTTCCTGCTGTTGC
At2g20420	GGATGGCTGAACTGGTTGAAGG	GAGAATGTGGATAGGACTGTGAACTG
FAD12	GACCGATTCGCTTGCCACTTC	GACATCAGAAAGGAGGATTTGAGACC
LTA2	TGCTGCCGCTGCTCCTAC	TGCTGCTTCGCCAACTTCTTC
RGS1	GGTTGTCTTCCTATTCTTTGTGGTTTG	TTCCTGAATCTCTTCTTGATGCTTGG
RPV1	CGAAGAATCTGGAGCCTGTTATGAC	TGCGGAACCTGGACCTAAGC
SCOA	CCTCAAGCATAGCCACTCCAATC	CACAAGACAAGATCAAGACCCTCAG
KAS1	AGCACCAAGGCAATGTCCAATC	CTGTAAAGAAGGTATTCAAGAGCACTG
PAS2A	CCTCCCTCCTCTAGCCATTTCTC	AGGACACGGCTTGGAACTCTC
At3g13930	TCTTCAACAGTTATGGCAATCACCTC	GCGACAGTAGAGATGGAATGTATGG
